# Proteomic Analysis of Stage-II Breast Cancer from Formalin-Fixed Paraffin-Embedded Tissues

**DOI:** 10.1155/2016/3071013

**Published:** 2016-03-24

**Authors:** Naif Abdullah Al-Dhabi, Srisesharam Srigopalram, Soundharrajan Ilavenil, Young Ock Kim, Paul Agastian, Rajasekhar Baaru, Kannan Balamurugan, Ki Choon Choi, Mariadhas Valan Arasu

**Affiliations:** ^1^Department of Botany and Microbiology, Addiriyah Chair for Environmental Studies, College of Science, King Saud University, P.O. Box 2455, Riyadh 11451, Saudi Arabia; ^2^Grassland and Forage Division, National Institute of Animal Science, RDA, Seonghwan-eup, Cheonan-si, Chungnam 330801, Republic of Korea; ^3^Department of Medicinal Crop Research, Rural Development Administration, Eumseong, Chungbuk 369-873, Republic of Korea; ^4^Research Department of Plant Biology and Biotechnology, Loyola College, Nungambakkam, Chennai, Tamil Nadu 600034, India; ^5^Proteomics Division, Discovery Research, Dr. Reddy's Laboratories Ltd., Miyapur, Hyderabad 500049, India; ^6^Sharmila Institute of Medicinal Products Research Academy, Thanjavur 613007, India

## Abstract

Breast cancer is the most frequently occurring disease among women worldwide. The early stage of breast cancer identification is the key challenge in cancer control and prevention procedures. Although gene expression profiling helps to understand the molecular mechanism of diseases or disorder in the living system, gene expression pattern alone is not sufficient to predict the exact mechanisms. Current proteomics tools hold great application for analysis of cancerous conditions. Hence, the generation of differential protein expression profiles has been optimized for breast cancer and normal tissue samples in our organization. Normal and tumor tissues were collected from 20 people from a local hospital. Proteins from the diseased and normal tissues have been investigated by 2D gel electrophoresis and MALDI-TOF-MS. The peptide mass fingerprint data were fed into various public domains like Mascot, MS-Fit, and Pept-ident against Swiss-Prot protein database and the proteins of interest were identified. Some of the differentially expressed proteins identified were human annexin, glutathione S-transferase, vimentin, enolase-1, dihydrolipoamide dehydrogenase, glutamate dehydrogenase, Cyclin A1, hormone sensitive lipase, beta catenin, and so forth. Many types of proteins were identified as fundamental steps for developing molecular markers for diagnosis of human breast cancer as well as making a new proteomic database for future research.

## 1. Introduction

Breast cancer is common lethal cause of malignancy among women around all the countries. Early detection of breast cancer facilitates the diagnosis and treatment prior to metastasis [1]. Despite remarkable development in new medicine findings and therapies for breast cancer during previous decades, no significant treatment methods are accessible for cancer-affected people with invasive and metastatic breast cancer. In this stage, the patients have less responses to cancer therapy due to recurrence properties of cancer [2]. The incident of breast cancer is increasing in India and also this is the second most common cancer in rural Indian females [3]. Furthermore, Indian women with breast cancer do not get medical assistance at early stage due to financial constraints, illiteracy, and lack of awareness. It is barely shocking that major elements of people with breast cancer in India do not get proper treatment at different stages of tumor progression [4]. The developed countries with small amount of global population reported approximately 50% of breast cancer diagnosed worldwide [5]. Far eastern and southeast Asian countries reported lowest breast cancer incidence [4]. Developing countries in Asia accounted for steadily increasing breast cancer population because of health care burdens and they are expected to have the highest rate of breast cancer incidence in the coming decades. Particularly, over 100000 breast cancer cases are accounted to be diagnosed every year in India [6, 7].

Mammography plays significant role in diagnosing breast cancer; however, the tumor size less than 0.5 cm remains undetectable by this model. The survival rate of this cancer patient is majorly associated with tumor conditions. Subsequently tumor identification of early stage-I has 98% of 5-year survival rate; stage-II tumors have 85%, stage-III tumors have 60%, and stage-IV has around 20% of 5-year survival rate for this cancer. In general, breast cancer has 5-year survival rate of 80% approximately with 207,090 cases and 39,840 deaths happening in women in America in the year of 2010. In 2015, approximately 231840 cases had breast cancer; among them 40290 people are going to die in the United States [8]. Breast cancer detection at early stage has treatment such as surgical resection with removal of axillary lymph nodes, radiation therapy, chemotherapy [3], and hormone therapy [9]. Though there is some notable improvement in treatment of breast cancer, the absence of biomarkers in serum/plasma causes delays in early identification of breast cancer [10]. This information signifies the requirement of new techniques in early diagnosis of breast cancer researches to the society. Stage-I patients have nine times more likelihood of staying healthy for ten years as compared with advanced periods [11]. This cancer becomes complex through invasive stage due to many changes in molecular level; it stimulates cell proliferation and genetic instability. This heterogeneity creates different subgroups that cause different clinical and therapeutical responses. Hence, it is necessary to determine the molecular structure including protein markers which are responsible for the diseases. These increase the rate of advanced stage analysis of the disease, therapeutical response, and the relapses after the therapy and the differences that are atypical to disease and person [12].

Hence, it is very urgent to make novel diagnostic methods for early stage detection of this cancer, which provide a new way to reduce this cancer related mortality [13, 14]. The sequencing of human genome has given a new way for the tremendous revolution in biology and medical field today [15]. The number of emerging and powerful technologies within functional genomics and proteomics combined with bioinformatics tools accelerates the application of basic discoveries in clinical practice [16].

Recent improvement in the field of molecular genetics, particularly proteomics, paved the way for improving the drug development and clinical trial procedure [17]. In addition, proteomics provides tools for investigating abnormal molecular changes in cancer tissues and it gives new insights into developing new reagents of understanding all the stages of cancer conditions [18]. Furthermore, proteomics also provides tools for drug discovery [19]. Although proteomics studies have been initiated in the area of breast cancer research in other world populations, limited studies are reported till date in Indian population. Keeping this in view, we at Dr. Reddy's Laboratories initiated breast cancer proteomics to understand the subtle changes in protein patterns in cancer patients using the proteomics technology. The ultimate aim of the project is to identify breast cancer biomarkers.

## 2. Materials and Methods

Breast cancer tissues were received from patients undergoing mastectomy at Mehdi Nawaz Jung (MNJ) cancer hospital, Hyderabad, India. All patients were found to be serologically negative for HBS Ag and HIV. The permission was gotten from all patients (20) in the age group of 24–60 years. All selected patients were suffering from infiltrating ductal cell carcinoma at the stage-II progression. Small pieces of samples were sliced and used for histology analysis [20]. Histopathological analysis of all the samples was performed at L. V. Prasad Eye Institute, Hyderabad, India, and the cancerous tissue was separated from normal tissue.

### 2.1. Sample Preparation

Histopathological separated normal and tumor tissues were homogenized in Bio-Rad's sample extraction reagent 2 buffers. The protein was estimated by Bio-Rad's RCDC method. Equal amount of protein was dissolved in 300 *μ*L Bio-Rad's rehydration buffer and loaded into 17 cm IPG strips (pH ranges from 4 to 7). Then the strips were focused using the PROTEAN IEF (Isoelectric Focusing) cell kit.

### 2.2. Image Analysis

The gels were scanned on Bio-Rad's G800 densitometry scanner. The images were analyzed for differential expression between normal and tumor gels by Bio-Rad's PDQuest software.

### 2.3. In-Gel Digestion/Mass Spectrometry (MALDI-TOF) Analysis

The excised silver stained in-gel protein band was chopped into small pieces and transferred into Eppendorf tubes. A piece of protein-free acrylamide gel was taken in parallel as a negative control. Mass spectrometry (MALDI-TOF) analysis was performed [21].

## 3. Results

### 3.1. 2D Gel Analysis

**Figure 1:**
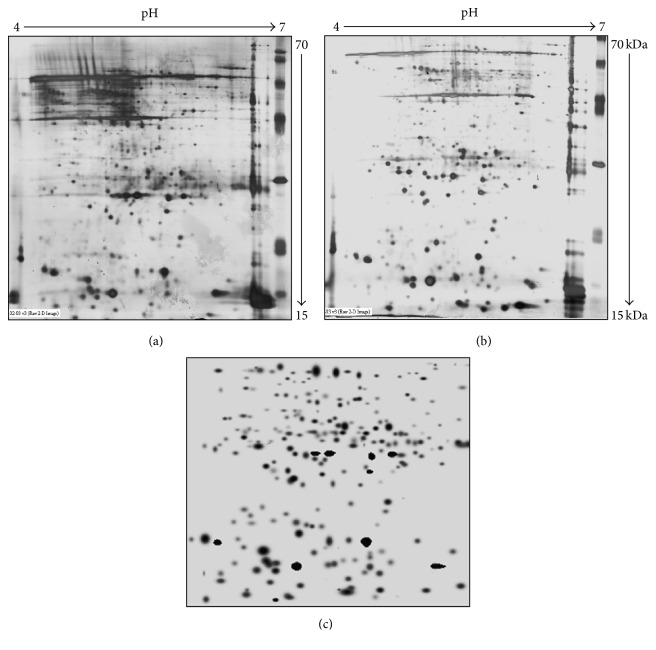
Differential proteins' expression in normal and breast cancer tissues analysis, by 2D gel electrophoresis. (a) Normal tissue. (b) Breast cancer tissue. (c) Master gel image.

The proteins of (400 *μ*g) histopathologically segregated normal and tumor breast tissue lysate were separated using 2D gel electrophoresis (17 × 20 cm) as represented in Figure 1. The images results exhibited many differentially expressed proteins in the breast cancer sample as compared with control tissues. The proteins with more than twofold average quantitative expressions between cancer and control tissues were considered as statistically regulated proteins. Among these, 29 spots and 6 spots were upregulated and downregulated in cancer condition, respectively, as compared with control tissues. Differential proteins were chosen for peptide mass fingerprinting analysis using MALDI-TOF.

### 3.2. MALDI-TOF Analysis

**Figure 2:**
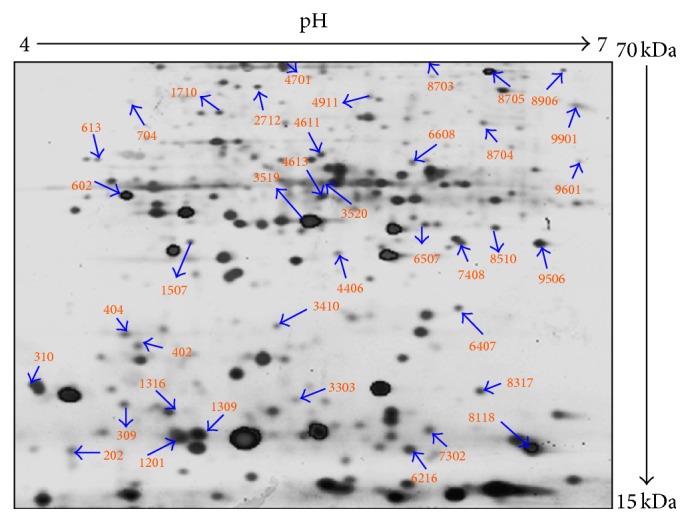
Image analysis of differentially expressed proteins in tissues by Bio-Rad PDQuest software with SSP numbers.

The proteins expression analysis by 2D gels in cancer and normal tissues was resolved in 12% SDS-PAGE and then stained. The selected protein spots were cut from the gels after image analysis (Figure 2 with SSP numbers). The differentially expressed proteins were identified based on the peptide mass fingerprints in cancer tissues as well as in control tissues (Table 1).

**Figure 3:**
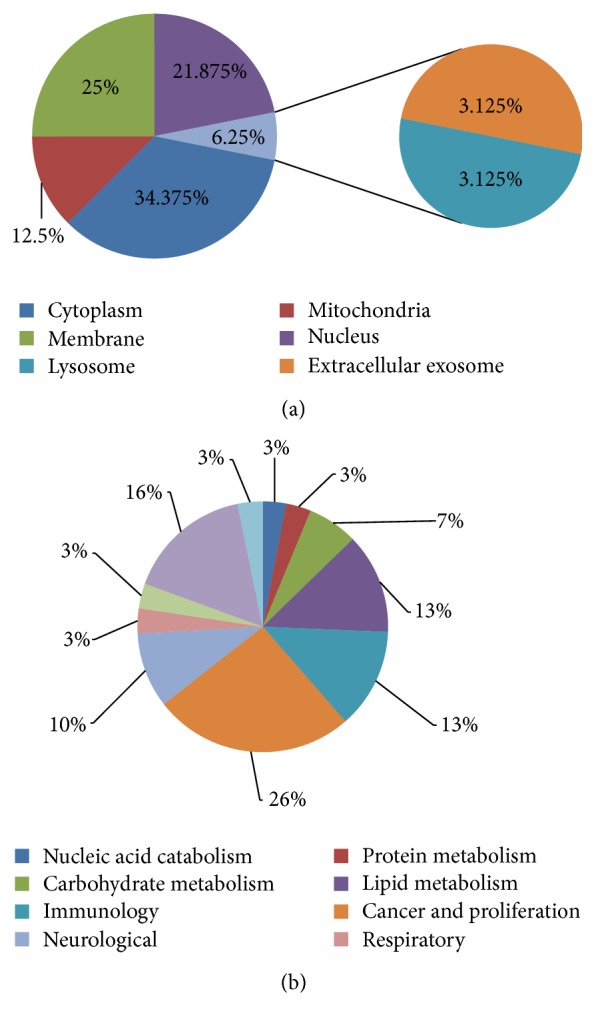
Classification of proteins based on their cellular component (a) and putative functions (b).

The identified proteins were categorized with their cellular component. Maximum numbers of proteins were found in the cytoplasm followed by the membrane, nucleus, mitochondria, and others (Figure 3(a)). Furthermore, these proteins were classified again based on the biological functions (Figure 3(b)).

## 4. Discussion

The rate of incidence and problems associated with cancer has been increasing over the last fifteen years for both men and women. Particularly in developed areas, the uterus and cervical cancers had the highest incidence in the last fifteen years among women populations. However, nowadays they are replaced by breast cancer. Classification of molecular events creates the main challenge in human breast cancer research. Achieving this goal is interrupted by the practical aspects of the application of improved methods to the microscopic premalignant and preinvasive stages of cancer [22]. The above factors are supported by the several evidences which propose that environmental pollution is a well-known etiological factor for breast cancers [23].

The present study focuses on the differential profiling of the breast cancer proteome, by comparing proteins using two-dimensional gel electrophoresis of both normal breast tissue and the tumor tissue. Some of the proteins identified are playing a crucial function in the disease progression and some are reported in the literature as tumor suppressor proteins. Human annexin significantly regulates tumor progression by stimulating cell proliferation and differentiation process [24]. The expression of annexin in tumor tissue strengthens its role in tumor progression and in similar way our results outcome correlated. Furthermore, the expression of annexin in tumor tissue strengthens its role in tumor progression and clinical features of breast cancer [25]. Similarly, human glutathione S-transferase is also known to be expressed in a variety of tumor tissues and breast cancer is one among them. This enzyme exerts the detoxification of cancer promoting reactive metabolites. The genetic polymorphisms of glutathione S-transferase (GST) enzyme T1, M1, P1, and A1 in Thai breast cancer patients related to progression of breast cancer. Therefore the increased expression of GST in this research confirms its role in tumor prognosis and supports the previous reports [26, 27].

Vimentin is another protein that has been identified in this study. Vimentin commonly showed more expression in myoepithelial cells with low molecular keratins as the trademark of glandular breast cells [28, 29]. The expression of vimentin in this study correlates with its possible role in cancer invasiveness and therefore proposes the different hypothesis in which vimentin in breast cancer could derive from breast progenitor cells with bilinear differentiation potential [30]. Enolase-1 is known to be a multifunctional enzyme and has a main role in glycolysis and it plays a part in different processes like growth control, hypoxia tolerance, and allergic responses [31]. Enolase-1 expressions in glycolytic process at hypoxia favor the tumor cells to solve their energy requirements. Consequently enolase-1 increases the survival rate, proliferation, and the invasive and metastatic ability of the tumor cells [32]. Hence, the expression of enolase-1 in our study validates its role in breast cancer cell energy requirements. Furthermore, downregulation of enolase-1 improves the cellular sensitivity to the radiation therapy and it might be the target for drug development for breast cancer [33].

Hormone sensitive lipase (HSL) plays potential role in lipogenesis, degradation, and its catabolism [34]. The expression of HSL in breast tissue proves its differential expression in breast cancer and its survival rate [35]. Conversely *β*-catenin is involved in transcriptional regulation of Wnt signaling cascade [36]. In our study, this protein's expression in breast tissue is related to invasive lobular breast carcinogenesis. The activation of *β*-catenin/Wnt signaling pathway is associated with low clinical output and is unlikely to be regulated by *β*-catenin encoding gene mutations in breast cancer [37]. On the other hand, cell cycle regulatory pathways play an important role in estrogen related breast cancer cell growth, in which Cyclin A1 plays important role in tumor development. Alterations in vascular endothelial growth factor related cellular pathways resulted in high expression of Cyclin A1 in primary and metastatic breast cancer specimens [38]. Hence, our finding indicates that the expression of Cyclin A1 in tumor sample correlates with the involvement of various cell signaling pathways like cell cycle regulators and estrogen receptor signaling in breast cancer progression. Likewise other proteins listed in Table 1 have important role in tumor progression.

## 5. Conclusions

Analyzing cancer using proteomic approach shed significant light on the underlying mechanism that leads to cancer development. The results discussed in the present study relate the expressed proteins involvement in tumor and cancer development related cellular pathways like cell cycle, angiogenesis, and metastasis. Based on this research outcome we propose that differentially expressed proteins in cancerous condition could be fundamental steps for developing the markers and proteomic database for breast cancer diagnosis.

## Figures and Tables

**Table 1:** Differentially expressed proteins in cancerous tissues with SSP numbers.

S. numbers	SSP	Proteins name
(1)	202	P56851/epididymal secretory protein E3 beta precursor
(2)	309	Q59699 D-hydantoinase
(3)	310	P50336-00-01-00/splice isoform displayed
(4)	402	Human GST
(5)	404	Q9Y5H9 splice isoform
(6)	613	MUTS2 protein
(7)	704	Vimentin
(8)	1201	Q9Y276 splice isoform displayed
(9)	1309	Dehydroquinate synthase
(10)	1507	Q9NRC8 splice isoform displayed
(11)	1709	O32720 anti-sigma-F factor antagonist
(12)	1710	P51587 human splice isoform II displayed
(13)	2712	Kinesin like protein
(14)	3303	AAA52735 immunoglobulin alpha-1 chain fragment
(15)	3410	AX879017 NID *Homo sapiens*
(16)	4406	Q860R0 MHC class I b antigen
(17)	4611	Q13085 human acetyl-CoA carboxylase
(18)	4613	Splice isoform displayed
(19)	4613	O94805 BRG1-associated factor
(20)	4701	Epithelial-cadherin precursor
(21)	4911	CUL5 protein
(22)	6216	Q92817 envoplakin
(23)	6507	Annexin A1 (annexin I)
(24)	6608	Glutamate dehydrogenase (GDH)
(25)	7302	Q15828 cystatin M precursor (tumor suppressor)
(26)	7408	Q9VPI8 DNA binding transcription factor
(27)	8317	Q9Y6N7 human splice isoform I
(28)	8510	Q05469 hormone sensitive lipase
(29)	8703	Beta-catenin
(30)	8704	Enolase-1
(31)	8705	P78396 Cyclin A1
(32)	8906	Dihydrolipoamide dehydrogenase, mitochondrial precursor
(33)	9506	GTP binding protein
(34)	9601	Lipid phosphate phosphohydrolase 3
(35)	9901	Cathepsin L2 precursor (cathepsin V)

## Abbreviations

2D: Two-dimensional polyacrylamide gel electrophoresis
DTT: Dithiothreitol
GDH: Glutamate dehydrogenase
GST: Glutathione S-transferase
HSL: Hormone sensitive lipase
MALDI-TOF-MS: Matrix-assisted laser desorption/ionization time-of-flight mass spectrometry
MNJ: Mehdi Nawaz Jung
NH4HCO3: Ammonium bicarbonate.
